# *Salmonella* Typhimurium environmental reduction in a farrow-to-finish pig herd using a live attenuated *Salmonella* Typhimurium vaccine

**DOI:** 10.1186/s40813-021-00222-1

**Published:** 2021-07-23

**Authors:** Peter van der Wolf, Maaike Meijerink, Emile Libbrecht, Gerrit Tacken, Emile Gijsen, Kathrin Lillie-Jaschniski, Verena Schüller

**Affiliations:** 1 Ceva Santé Animale BV, Naaldwijk, The Netherlands; 2 Ceva Santé Animale S.A., Brussel, Belgium; 3 Veterinary Practice “VarkensArtsenZuid”, Panningen, The Netherlands; 4 Ceva Tiergesundheit GmbH, Düsseldorf, Germany

**Keywords:** Salmonella, Swine, Pig, Vaccination, Eradication, Disease control, Risk factors, Salmonellosis, Zoonosis, Antimicrobial use

## Abstract

**Background:**

*Salmonella* Typhimurium is an important zoonotic pathogen in pigs, that can cause clinical disease. Many sow herds and finishing herds are infected with *Salmonella*, and therefore pose a threat for the contamination of pork and pork products and ultimately consumers.

**Case presentation:**

This case study describes a farrow-to-finish pig herd, producing its own replacement gilts, which had experienced clinical outbreaks of salmonellosis since 2002. Outbreaks were characterised by profuse diarrhoea, dead pigs and high antimicrobial use (colistin sulphate). The aim of this study was to see whether using vaccination of sows and piglets with Salmoporc®, a live attenuated *Salmonella* Typhimurium vaccine, in combination with standard hygienic precautions, it was possible to reduce *Salmonella* Typhimurium to below the bacteriological detection limit. Monitoring of the presence of *Salmonella* was done using a total of 20 pooled faecal, sock and dust samples per herd visit in the period from September 2016 to October 2020. Within the first 10 months after the start of vaccination in August 2016, there was a rapid reduction in clinical symptoms, antimicrobial usage and the number of *Salmonella*-positive samples. During the winters of 2017/2018 and 2018/2019 the number of positive samples increased again, however with minimal need to use antimicrobials to treat the affected animals. In July 2019, only two samples from a corridor were positive. In September and November 2019 and in October 2020 all three samplings were completely negative for *S*. Typhimurium.

**Conclusions:**

This case, together with other longitudinal studies, can be seen as a proof of the principle that long term vaccination with a live attenuated *S.* Typhimurium vaccine can reduce the level of *S*. Typhimurium in the herd environment to very low levels within a farrow-to-finish herd initially suffering from clinical salmonellosis. Also, clinical symptoms indicating salmonellosis were no longer observed and antimicrobials to treat clinically diseased pigs were no longer needed.

## Background

*Salmonella* and particularly *Salmonella* Typhimurium (S. Typhimurium or ST) is an important zoonotic pathogen occurring worldwide [1–3], able to cause serious extra-intestinal disease in humans [4]. *Salmonella* Typhimurium, and it’s monophasic variant, is a pathogen in the pig industry that can infect and colonize pigs [5]. This can lead to enteritis, both acute and subclinical, accompanied by a reduction in average daily weight gain and an increase in feed conversion ratio. In some cases (per)acute mortality, mainly in growers and finishers, can occur in any age category [6–8]. Control of *Salmonella* is difficult and based on the control of many different risk factors within pig herds [9]. Relevant control factors include all-in/all-out procedures, internal and external biosecurity, rodent-, fly- and beetle control, thorough cleaning and disinfection, improved pig management practices (e.g. reduction of cross fostering of suckling piglets and mixing of piglets at weaning or at transfer to the grow/finishing units), the use of specialized feed formulations, the use of organic acids and vaccination and the control of concomitant infections like *Ascaris suum*, PRRSV, ileitis or dysentery [10–17].

Abundance of *Salmonella* in the environment and within pig farms makes a lasting *Salmonella* free status of pig farms a difficult goal. However, pig farms in Norway and Sweden are practically free of *Salmonella* (< 0.1% prevalence at herd level [18, 19]) and pig farms that use fermented liquid feed as a feeding system [20, 21] can remain free of *Salmonella* for at least 2 years [22] in a country with a high prevalence of *Salmonella* in sow herds [23]. For the control of *Salmonella* Typhimurium in pig herds, in addition to trying to control all possible risk factors, an option is to use vaccination as a tool to boost immunity in the pigs and consequently reduce both their susceptibility to infection and shedding after infection. Salmoporc® (Ceva Santé Animale, Libourne, France, formerly IDT Biologika GmbH, Dessau, Germany) is a lyophilizate of a genetically stable live double attenuated (histidine-adenine-auxotrophe) *Salmonella* Typhimurium strain (nr. 421/125). After dilution, one dose of 1 ml contains 5*10^8 to 5*10^9 colony forming units. In sows it is applied by subcutaneous injection, in piglets it is given twice orally by drench. Salmoporc® is registered in several European countries, including Belgium and The Netherlands. The claims of the vaccine are that in gilts and sows it will induce immunity and reduce shedding during lactation and in piglets it will induce active immunity resulting in a reduction in colonisation, shedding and clinical symptoms. As the vaccine is a live vaccine, even sub cutaneous instead of oral administration stimulates both humoral and cellular immunity and therefore able to protect against an enteric pathogen and reduce its shedding [24]. High humoral and cellular immunity in piglets is desirable to protect against early colonisation of newborn piglets, even though piglets are vaccinated from 4 days of live. Maternally derived immunity does not interfere with the effect of the vaccination of piglets, on the contrary, Rösler et al. showed that vaccinated piglets from vaccinated sows had significantly lower infection levels after challenge than vaccinated piglets from unvaccinated sows [25]. The effectiveness of Salmoporc® is well documented [26–29] for the reduction of colonisation and shedding of *Salmonella* Typhimurium, sometimes even in the unvaccinated progeny of vaccinated sows [30, 31]. However, to our knowledge, no studies exist that followed a vaccinated herd to the point where *Salmonella* Typhimurium could no longer be isolated from faecal and dust samples.

The aim of this study was, next to the reduction of clinical salmonellosis and antimicrobial use, to investigate whether it was possible to reduce *Salmonella* Typhimurium in a farrow-to-finish pig herd with a history of clinical salmonellosis to below the bacteriological detection limit, using vaccination of sows and piglets with Salmoporc®.

## Material and methods

### The farm

**Fig. 1:**
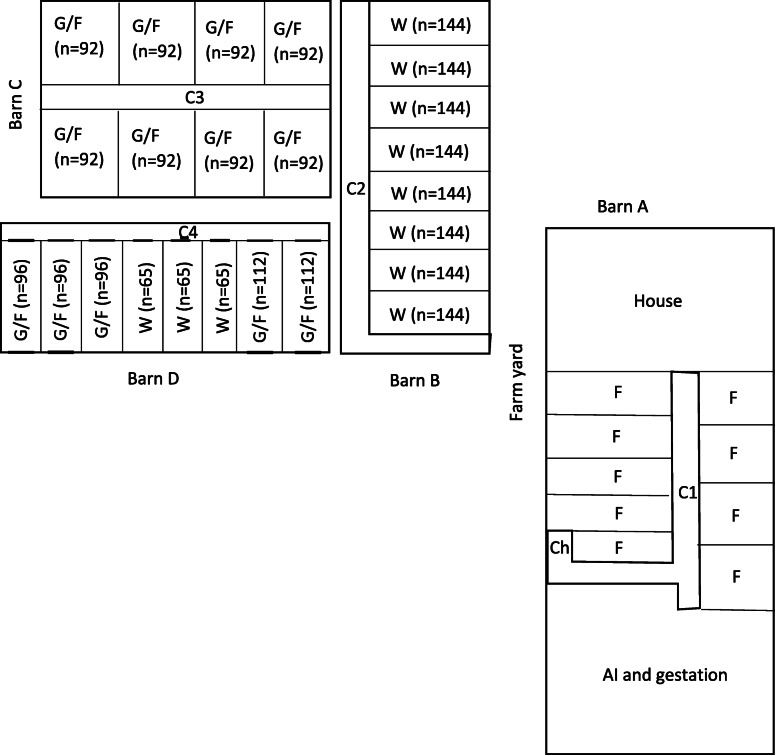
Layout of the trial farm. Ch: Changing room, C1 – C4: corridors, F: Farrowing, W: Weaned piglets, G/F: Grow/Finishers, number of animals per compartment given in brackets

The herd in this study is located in the north-eastern part of Belgium close to the Dutch border. The herd is a 260-sow multiplying herd, producing its own replacement gilts and finishing pigs. Sperm doses are brought in from a boar stud outside the farm. The herd is managed based on a five-week production cycle. The herd consists of 4 buildings (barns A – D, Fig. 1).

Replacement gilts (approx. 50–60) and grow/finishing pigs are mostly housed in separate compartments within the same barns (Fig. 1, C and D). Additionally, some of the growers (approx. 25 kg BW) are moved to an off-site finishing unit or are sold to other local farms (not included in this study). The herd is entered via a changing room located at the entrance of the sow barn (Fig. 1, Ch), where boots and overalls are supplied to visitors and there are hand washing facilities. There is no strict separation between clean and dirty areas, transport lorries for feed, slurry and farm supplies and visitors use the farmyard between the sow barn and the other barns (Fig. 1, farm yard). Boots are not changed and boot dips are not used between barns. To reach the barns of the gilts and the finishers (Fig. 1, C and D), one has to walk along the central corridor of the barn housing the weaned piglets (Fig. 1, C2). Compartments are managed on an all-in/all-out basis. Empty compartments are cleaned by high pressure cleaner with cold water and sanitised with a disinfectant containing glutaraldehyde/quaternary ammonium components according to the manufacturer’s instructions (Megades Novo®, Schippers, The Netherlands). The floors in gestation, AI-centre and the compartments with weaned piglets are fully slatted. Floors in the finishing barns are made of concrete and partly slatted within each pen. Pen separations do not prevent exchange of faeces and/or urine between pens. Flies and mice are present, but not in large numbers. Rodent and fly control is done by the farmer himself. All pigs are fed compound pelleted feed suitable for their age group or production cycle. No routine preventive antimicrobials were used in this farm, however quite a lot of antimicrobials were used especially in weaned piglets for the (metaphylactic) treatment of infections caused by *Streptococcus suis,* 76.3% of all antimicrobials used in the years 2014–2020. In Table 1 the total added antimicrobial treatment index values as defined by AMCRA in Belgium (BD100, www.amcra.be/nl/analyse-antibioticagebruik/) is given for the years 2014–2020, per pig category treated and per antimicrobial category. Polymyxins, specifically used to treat clinical salmonellosis in this farm, were never used in sows or suckling piglets. Weaned piglets and grow/fattening pigs were treated with polymyxins orally via the drinking water.

**Table 1:**
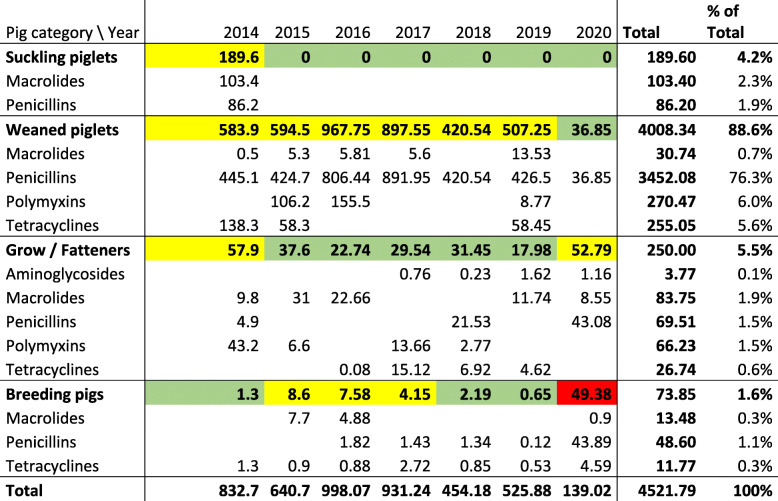
Total added antimicrobial treatment index values applicable in Belgium (BD100) per year, presented for the years 2014–2020, per pig category treated and per antimicrobial category. Colours: green: acceptable level of use, yellow: attention level, red: action level (AMCRA, Belgium)

### Salmonella history of the farm

A chronological history of the development of the *Salmonella* situation on the herd is given in Table 2 in the period from 2002 to August 2016 when vaccination was started.

**Table 2:** Chronological history of the *Salmonella* situation at the farm. Presented are the date, the subject and the source of the information in the period from 2002 to 2016

November 2002	The earliest mention of diarrhoea, describing considerable amounts of grey, yellow and sometimes black diarrhoea	Visit report of the veterinarian
September 2004	Confirmation of anti-*Lawsonia intracellularis* serum IgG in pigs over 50 kg of body weight and advises medication with Tylosin	Visit report of the veterinarian
April 2005	Confirmation of *Salmonella* in faecal samples of gilts of 40–60 kg of body weight and advised treatment with Enrofloxacin (Baytril®) and colistin sulphate. The appearance of runts in pigs over 30 kg of body weight is mentioned as a problem.	Visit report of the veterinarian
October 2009	High S/P-ratio values in the *Salmonella* ELISA, especially in heavy finishers > 80 kg BW (4 out of 7 pigs sampled had S/P-ratio values > 2, all 7 pigs were positive at cut-off 0.6)	Laboratory DGZ Vlaanderen vzw
	Later reports mention different S/P-ratio values, sometimes high, sometimes low, in pigs of 40 kg body weight and upwards	Laboratory DGZ Vlaanderen vzw
2012–2015	All most all reports mention *Salmonella* as a problem	Visit reports of the veterinarian
October 2012	The *Salmonella* expert from the Animal Health Service Flanders visited the herd to provide advice. Different acids (based on formic acid or coated butyrate) were tried through feed and/or drinking water, however concentrations nor duration of treatment nor pig category were specified or recorded.	DGZ Vlaanderen vzw
October 2012	A faecal sample from sows and gilts / finishers tested positive for *Salmonella* Typhimurium O5+ which was sensitive to all tested antimicrobials including colistin.	DGZ Vlaanderen vzw, typing by CODA-CERVA
March 2013	Two heavy finishers (104 and 106 kg body weight respectively) were submitted for post-mortem examination and enteritis caused by *Salmonella* Typhimurium O5+ was confirmed	DGZ Vlaanderen vzw, typing by CODA-CERVA
October 2013	Seven pigs died of which 4 were presumed to have died of salmonellosis. Colistin sulphate is used regularly to treat for diarrhoea.	Visit report of the veterinarian
April 2014	The report mentions the options of vaccinating for *Salmonella* and the use of butyric acid in the feed.	Visit report of the veterinarian
2014–2016	As soon as diarrhoea appeared, antimicrobials containing colistin sulphate (Colistine-mix 1.2 milj. I.U./g, 1 kg during 2015, dose 5 mg/kg BW via drinking water for 5–7 days and Promycine Pulvis 4800 IE/mg, 1 kg, dose 100,000 I.U. per kg BW for 3–5 days via the drinking water after 2015) were used to treat the pigs.	AMCRA Belgium
May 2016	Salmonellosis was confirmed after typing of 4 isolates from diarrhoea samples as *Salmonella* Typhimurium O5+ which were sensitive to all tested antimicrobials including colistin.	DGZ Vlaanderen vzw, typing by CODA-CERVA
October 2016	Necropsy of 3 non-vaccinated finishing pigs (80, 80 and 84 kg body weight), showing moderate congestion of the mesenteric lymph nodes, dilated jejunum with yellow-brown fluid content including some *Ascaris suum* worms, very fluid yellow-brown content of the large intestine, confirmed they died of salmonellosis caused by *S.* Typhimurium O5+ which was sensitive to all tested antimicrobials including colistin.	DGZ Vlaanderen vzw, typing by CODA-CERVA

### Other vaccinations

In general, the sows are mass vaccinated against PRRSV four times per year; *E. coli* at the end of gestation; and Parvo / Erysipelas during lactation. The replacement gilts are vaccinated against *Mycoplasma hyopneumoniae* at 17 days of age; ileitis (*Lawsonia intracellularis*) at 20 days of age; PRRSV together with the sows and Parvo / Erysipelas during the rearing period from 3 to 7 months of age. An outbreak of PRRSV occurred in the sows in 2018, which lasted for approx. 6 months.

### Choice of diagnostic method

Several methods can be used to assess the *Salmonella* status of pigs and pig farms. Serology based on ELISAs [32] can be used on a herd level or national level or even in small studies to assess the effect of Salmonella interventions [29, 33], but ELISAs cannot differentiate between antibodies in response to infection with field strains or vaccination with Salmoporc [34], making this method unsuitable for our study. Another option would be to look at mesenteric lymph nodes at the slaughterhouse, however, cross contamination during the period of transport, holding and slaughter cannot be ruled out and would make genetic differentiation of field strains necessary to make sure that the collected field strains originated from this farm, which is labour intensive, costly and already done before [35]. Therefore we chose for collecting faecal and dust samples from the pens and corridors in the farm itself, also because a reduction of shedding are claims of the vaccine for both sows and piglets.

### Sampling procedure of pooled faecal samples, sock samples, dust samples

Pooled faecal samples were collected in 50 ml plastic containers. From every pen within a compartment at least two faecal samples, taken from the floor of the pens, were added to the pool. One pooled sample represented one compartment. The plastic containers were labelled with an identification number corresponding to the sampling protocol. Sock samples were collected by covering each boot with a plastic overshoe (boot cover, transparent, Covetrus, The Netherlands) at the entrance of the compartment. A sock (non-skid, non-conductive, blue shoe covers, Henry Schein, Covetrus, The Netherlands) was put over one plastic overshoe to collect faecal material from the floor of all pens within the compartment. With this sock we purposefully stepped into faecal material accumulated in each pen, usually along the walls and in the corners of the pen. One sock was collected per compartment. When sampling corridors using these socks, also we purposefully stepped in any faecal material present. The socks were placed in individual plastic bags which were closed by tying a knot in the bag. Each bag was labelled with an identification number corresponding to the sampling protocol. Dust samples were collected wearing non-sterile gloves (lightly powdered latex examination gloves, Henry Schein, Covetrus, The Netherlands) and wiping as many surfaces as possible, both at animal level e.g. driving board, shovels, boots, walls, doors and higher, e.g. window sills, the top of pen separations and feeders, feed pipelines, ventilation shafts, in either a compartment or a corridor while collecting the sock and pooled faecal samples. For the dust wipes we used synthetic dust cloth (dry Swiffer® wipes or comparable local wipes from Action shop), one wipe per compartment or corridor [36]. The dust wipes were handled like the socks. All samples were shipped cooled by overnight courier to the laboratory. All samplings were carried out by a trained experienced person employed by either IDT Animal Health or after July 1st, 2019 Ceva Santé Animale.

### Sampling scheme of pooled faecal samples, sock samples, dust samples

Samplings were done every 4 weeks from August 2016 till April 2018. Since then, samplings were done every 8 weeks till November 2019 and the last one in October 2020.

Three different sampling schemes (I, II, III) were used for collecting pooled faecal, sock and dust samples, due to an improvement of the sampling scheme during the study [36]. The first change was to stop collecting pooled faecal samples. Instead, a sock and a dust sample was collected from each compartment or corridor. The change from II to III was that in compartments with pigs, no more dust swabs were taken, but more compartments were sampled with socks per visit. Sampling Scheme II was implemented starting November 2016 and samplings Scheme III starting May 2017 until the end of the trial. The type and number of samples taken at each herd visit are listed in Table 3 in the results. Slight variations on these sampling schemes were sometimes necessary due to the circumstances at the time of sampling, for example if there were no piglets present in the farrowing compartments due to the five-week farrowing batch system.

**Table 3:** Bacteriological results by sampling date and year and sample type per animal category and per location. C1, C2, C3, C4: corridors in barns A – D respectively, AI: artificial insemination centre (see Fig. 1). Sample types: S/D/P: combined sock sample, dust wipe and pooled faecal sample from the same compartment, S/D: sock sample and dust wipe from the same compartment or corridor, S: sock sample, . or -: not done. Bacteriological results for combined samples per compartment or corridor are presented as xxx/xxx/xxx or xxx/xxx, results of separate compartments are separated by, (comma). *: significant decrease since previous high number of positive samples (Fisher exact test, *P* < 0.05), #: significant increase since previous low number of positive samples (Fisher exact test, *P* < 0.05), field = *S*. Typhimurium field strain, ST = undifferentiated *S*. Typhimurium., group C = *Salmonella enterica* serogroup C isolate, vac = Salmoporc® vaccine strain, neg = negative

		C1	C2	C3	C4	Farrowing	AI and gestation	Weaned piglets barn B	Weaned piglets barn D	Grow finishers barn C	Grow finishers barn D	Positive and Total Salm samples	Remarks
Aug-16	location	.	1 S/D	1 S/D	.	1 S/D/P	1 S/D/P	1 S/D/P	.	1 S/D/P, 2 S/D	.		
	Salm result	.	neg/neg	field/field	.	neg/field/neg	neg/neg/neg	neg/neg/neg	.	neg/neg/neg, 2 field/field	.	7 / 20	1 G/F in barn C treated with colistin was ST-field positive
Sep-16	location	.	1 S/D	1 S/D	.	1 S/D/P	1 S/D/P	1 S/D/P	.	1 S/D/P, 2 S/D	.		
	Salm result		neg/neg	field/field		vac/vac/field	field/vac/neg	vac/neg/neg		field/field/field, field/neg, field/group C		10 / 20	Weaned piglets were being treated with amoxycillin
Okt-16	location	1 S/D	1 S/D	1 S/D	1 S/D	1 S/D/P	1 S/−/P	1 S/D/P	.	1 S/D/P	.		
	Salm result	ST/ST	neg/neg	ST/ST	ST/neg	ST/ST/neg	ST/./neg	ST/ST/neg	.	ST/neg/ST	.	12 / 19	Isolates were not differentiated between field and vaccine strain
Nov-16	location	.	1 S/D	1 S/D	.	1 S/D	1 S/D	1 S/D	.	5 S/D	.		Stopped pooled faecal samples
	Salm result	.	neg/neg	neg/neg	.	vac/vac	neg/vac	vac/neg	.	3 neg/neg, 2 field/field	.	4 / 20*	
Dec-16	location	1 S/D	1 S/D	1 S/D	.	1 S/D	1 S/D	1 S/D	.	3 S/D	.		
	Salm result	vac/neg	vac/neg	field/field	.	neg/neg	vac/vac	vac/vac	.	2 vac/neg, field/field	.	4 / 18	
**Total 2016**												**37 / 97 (38%)**	
Jan-17	location	.	1 S/D	1 S/D	.	1 S/D	1 S/D	1 S/D	.	5 S/D	.		
	Salm result	.	neg/neg	field/field	.	field/neg	field/field	vac/vac	.	neg/neg, field/neg, 2 field/field, neg/field	.	11 / 20#	1 G/F non-vaccinated was field/field-pos. 1 G/F with old F had diarrhoea: field/field
Feb-17	location	1 S/D	1 S/D	1 S/D	.	1 S/D	1 S/D	1 S/D	.	4 S/D	.		
	Salm result	neg/vac	vac/neg	neg/neg	.	field/vac	neg/vac	vac/vac	.	2 neg/neg, neg/vac, neg/field	.	2 / 20*	
Apr-17	location	.	1 S/D	1 S/D	.	1 S/D	1 S/D	2 S/D	.	4 S/D	.		
	Salm result	.	vac/neg	neg/field	.	neg/vac	vac/vac	vac/neg, vac/neg	.	2 vac/neg, neg/neg, field/neg	.	2 / 20	1 G/F in barn C had diarrhoea: field/neg
Mei-17	location	.	1 S/D	1 S/D	.	2 S	1 S/D	3 S	3 S	6 S	.		
	Salm result	.	neg/neg	neg/field	.	vac, vac	neg/vac	3 vac	2 vac, 1 neg	5 neg, 1 vac	.	1 / 20	Changed sampling scheme: no dust sample in compartments
Jun-17	location	.	1 S	1 S/D		1 S	1 S	2 S	2 S	6 S	1 S		
	Salm result	.	vac	neg/neg		vac	vac	vac, field	2 vac	4 vac, 2 neg	neg	1 / 16	
Jul-17	location	.	1 S/D	1 S/D	1 S/D	2 S	1 S	3 S	2 S	4 S	2 S		
	Salm result	.	neg/neg	neg/neg	neg/neg	vac, vac	vac	3 vac	neg, vac	3 neg, 1 vac	neg, vac	0 / 20	
Aug-17	location	.	1 S/D	1 S/D	1 S/D	1 S	1 S	3 S	2 S	4 S	3 S		
	Salm result	.	neg/vac	neg/field	neg/neg	neg	vac	3 vac	neg, vac	3 vac, 1 field	neg, 2 vac	2 / 20	
Sep-17	location	.	1 S/D	1 S/D	1 S/D		1 S	3 S	2 S	5 S	2 S		
	Salm result	.	neg/neg	neg/neg	neg/neg		neg	3 vac	vac, neg	3 neg, 1 vac, 1 field	2 neg	1 / 19	
Okt-17	location	.	1 S/D	1 S/D	1 S/D			4 S	2 S	5 S	2 S		
	Salm result	.	neg/neg	field/neg	neg/neg			3 vac, neg	2 vac	2 neg, 2 vac, 1 field	vac, field	3 / 20	1 G/F in barn C had diarrhoea: field
Nov-17	location	.	1 S/D	1 S/D	1 S/D	2 S	1 S	2 S	3 S	5 S	1 S		
	Salm result	.	neg/neg	field/neg	field/field	2 neg	field	vac, field	2 field, neg	4 field, neg	field	12 / 20#	3 G/F in barn C had diarrhoea and were treated with colistin, pos for field strain
Dec-17	location	.	1 S/D	1 S/D	1 S/D	2 S	2 S	2 S	2 S	5 S	1 S		
	Salm result	.	vac/neg	neg/neg	neg/field	2 vac	2 neg	2 vac	2 vac	vac, 2 neg, 2 field	neg	3 / 20*	
**Total 2017**												**38 / 215 (17.7%)**	
Jan-18	location	.	1 S/D	1 S/D	1 S/D	2 S	1 S	2 S	2 S	5 S	2 S		
	Salm result	.	neg/neg	neg/field	neg/field	neg, vac	vac	2 vac	2 vac	3 vac, 2 field	2 field	6 / 20	2 G/F in barn D had diarrhoea and were treated with colistin
Feb-18	location	.	1 S/D	1 S/D	1 S/D	1 S	1 S	3 S	2 S	5 S	2 S		
	Salm result	.	vac/vac	neg/neg	neg/neg	vac	neg	2 field, 1 vac	vac, field	field, vac, 3 neg	2 field	6 / 20	
Mrt-18	location	.	1 S/D	1 S/D	1 S/D	1 S	1 S	3 S	2 S	5 S	2		
	Salm result	.	vac/vac	vac/neg	vac/vac	vac	neg	3 vac	neg, vac	3 neg, 2 vac	neg, field	1 / 20	1 G/F in barn D with some diarrhoea, pos for field strain
Apr-18	location	.	1 S/D	1 S/D	1 S/D	1 S	1 S	3 S	2 S	5 S	2 S		
	Salm result	.	vac/vac	neg/neg	field/neg	neg	neg	vac, 2 neg	2 neg	4 neg, 1 vac	vac, field	2 / 20	1 G/F in barn D with some diarrhoea, pos for field strain
Jun-18	location	.	1 S/D	1 S/D	1 S/D	1 S	1 S	2 S	2 S	5 S	3 S		
	Salm result	.	neg/vac	neg/neg	neg/neg	vac	vac	2 neg	2 vac	3 vac, 2 neg	field, vac, neg	1 / 20	1 G/F in barn D with diarrhoea in one pen, pos for field strain
Aug-18	location	.	1 S/D	1 S/D	1 S/D	1 S	1 S	2 S	3 S	5 S	2 S		
	Salm result	.	vac/vac	neg/neg	field/neg	field	neg	2 vac	3 neg	4 neg, 1 vac	field, vac	3 / 20	1 G/F in barn C had some diarrhoea: neg, 1 G/F in barn D was also pos in jun-18
Okt-18	location	.	1 S/D	1 S/D	1 S/D	1 S	1 S	3 S	2 S	5 S	2 S		
	Salm result	.	neg/vac	field/field	neg/neg	neg	neg	2 vac, 1 neg	neg, field	4 field, neg	field, neg	8 / 20#	2 G/F in barn C some diarrhoea, both field strain pos
Dec-18	location	.	1 S/D	1 S/D	1 S/D	1 S	1 S	3 S	2 S	5 S	2 S		
	Salm result	.	neg/neg	neg/neg	field/neg	neg	field	3 neg	2 neg	1 vac, 4 field	neg, field	7 / 20	2 G/F in barn C some diarrhoea, both field strain pos
**Total 2018**												**34 / 160 (21.3%)**	
Feb-19	location	.	1 S/D	1 S/D	1 S/D	1 S	1 S	3 S	2 S	5 S	2 S		
	Salm result	.	neg/neg	field/neg	neg/neg	vac	neg	3 vac	2 vac	4 vac, 1 field	neg, field	3 / 20	1 G/F in barn C was also pos in dec-18
Apr-19	location	.	1 S/D	1 S/D	1 S/D	1 S	1 S	3 S	2 S	5 S	2 S		
	Salm result	.	vac/neg	neg/neg	neg/neg	field	neg	2 vac, 1 neg	2 neg	2 field, 3 neg	2 neg	3 / 20	
May-19	location	.	1 S/D	1 S/D	1 S/D	1 S	1 S	2 S	2 S	6 S	2 S		
	Salm result	.	neg/neg	field/neg	neg/neg	neg	neg	2 vac	2 vac	4 neg, 1 vac, 1 field	2 neg	2 / 20	1 G/F in barn C had some diarrhoea: pos for field strain
Jul-19	location	.	1 S/D	1 S/D	1 S/D	1 S	1 S	2 S	3 S	5 S	2 S		
	Salm result	.	neg/neg	field/neg	vac/field	neg	vac	2 vac	2 neg, 1 vac	5 neg	2 neg	2 / 20	
Sep-19	location	.	1 S/D	1 S/D	1 S/D	1 S	1 S	4 S	2 S	4 S	2 S		
	Salm result	.	neg/neg	neg/neg	neg/neg	neg	neg	4 vac	1 vac, 1 neg	2 neg, 2 vac	2 neg	0 / 20*	1 G/F in barn C has some diarrhoea: neg
Nov-19	location	.	1 S/D	1 S/D	1 S/D	.	2 S	4 S	1 S	5 S	2 S		
	Salm result	.	neg/neg	neg/neg	vac/neg	.	vac, neg	4 vac	vac	5 vac	vac, neg	0 / 20	
**Total 2019**												**10 / 120 (8.3%)**	
Oct-20	location	.	1 S/D	1 S/D	1 S/D	1 S	1 S	3 S	2 S	6 S	1 S		
	Salm result	.	vac/vac	neg/neg	vac/neg	vac	vac	2 vac, 1 neg	2 neg	2 vac, 4 neg	neg	0 / 20	
**Total**												**119 / 612 (19.4%)**	

### Bacteriological analysis

Bacteriological analysis of the pooled faecal, sock and dust samples was performed by the Microbiological Institute of the Centre for Infectious Diseases of the Veterinary University of Hannover (Institut für Mikrobiologie, Zentrum für Infektionsmedizin, Stiftung TiHo Hannover), using a protocol that was validated in comparison with the ISO-standard 6579 1:2017 and was found to be more sensitive than the ISO-standard. Samples were inoculated into 225 ml buffered peptone water (BPW; Oxoid, Germany) and incubated for 18 h at 37 °C. One ml of this non-selective pre-enrichment was transferred to 8 ml tetrathionate brilliant green bile broth (TBG; VWR, Germany) and 0.1 ml was transferred to Rappaport Vassiliadis Soy broth (RVS; Oxoid, Germany). The selective liquid media were incubated for 24 h at 42 °C and then streaked on Oxoid Brilliance *Salmonella* agar. After 24 h at 37 °C plates were inspected for growth of typical purple colonies. Colonies were subcultured on non-selective Columbia sheep blood agar (Oxoid, Germany). Isolates were submitted to an in-house-PCR that can specifically identify *Salmonella* Typhimurium as well as non-specifically *Salmonella enterica ssp. enterica* and *Salmonella* genus based on Park et al. [37]. One isolate per sample was typed. Isolates only identified as *Salmonella enterica ssp. enterica* or *Salmonella* genus by PCR were further characterized by slide agglutination with O- and H-specific antisera (Sifin, Germany, *Salmonella* Serogroup A-E and Vi, Serogroup F-67, Serogroup B, Serogroup C, Serogroup D, Serogroup E, *Salmonella* Derby). The Salmoporc® vaccine stain was differentiated from field strains of *S.* Typhimurium by a microbiological test called “IDT Salmonella Diagnostic Kit” which contains a nutrient broth which lacks adenine and histidine in which the vaccine strain does not grow and field strains do. No antimicrobial resistance testing was done on the isolated field strains.

### The Salmoporc® vaccination scheme

Sows vaccinated for the first time were vaccinated twice with one dose (1 ml) 3 weeks apart, approximately 6- and 3-weeks ante partum (ap). Consecutive vaccinations were done with one dose (1 ml) at 3 weeks ante partum. Onset of immunity is 2 weeks after the second vaccination and duration of immunity is 24 weeks. Piglets were vaccinated twice orally, 1 ml each, starting from 4 days of age, the second dose 21 days later. No antimicrobials were administered 5 days before and after vaccination. If treatment for *Streptococcus suis* infection was necessary, the vaccination was delayed until 5 days after the treatment was stopped. Onset of immunity is 2 weeks after the second vaccination and the duration of immunity in piglets is 19 weeks.

The vaccination protocol started at the end of August 2016 and continued until the end of the trial in November 2019. Thereafter, sows and gilts were vaccinated according to SPC, but newborn piglets were drenched only once at 5 days after birth.

### Data management and analysis

Data entry and result analysis was done using Excel (Office Excel, Microsoft Office Professional Plus 2016). Data entry was checked by a second person and corrected where necessary. Data collected were sampling date, sample number, sample type (pooled, dust, sock), animal category (farrowing, pregnant sows, AI centre, weaned piglets, breeding gilts / growers-finishers), sampling location (compartment or corridor), *Salmonella* positive yes/no, *Salmonella* Typhimurium positive yes/no, *S.* Typhimurium field strain or vaccine strain, and which type of *Salmonella* it was when not S. Typhimurium (e.g. serogroup C). A compartment or corridor was considered positive if any sample from that location was positive for *Salmonella*. Chi-square statistics or Fisher exact test were carried out using the calculators on the website Social Science Statistics (https://www.socscistatistics.com/tests/chisquare/default2.aspx or https://www.socscistatistics.com/tests/fisher/default2.aspx).

## Results

### Clinical salmonellosis and antimicrobial use

**Fig. 2:**
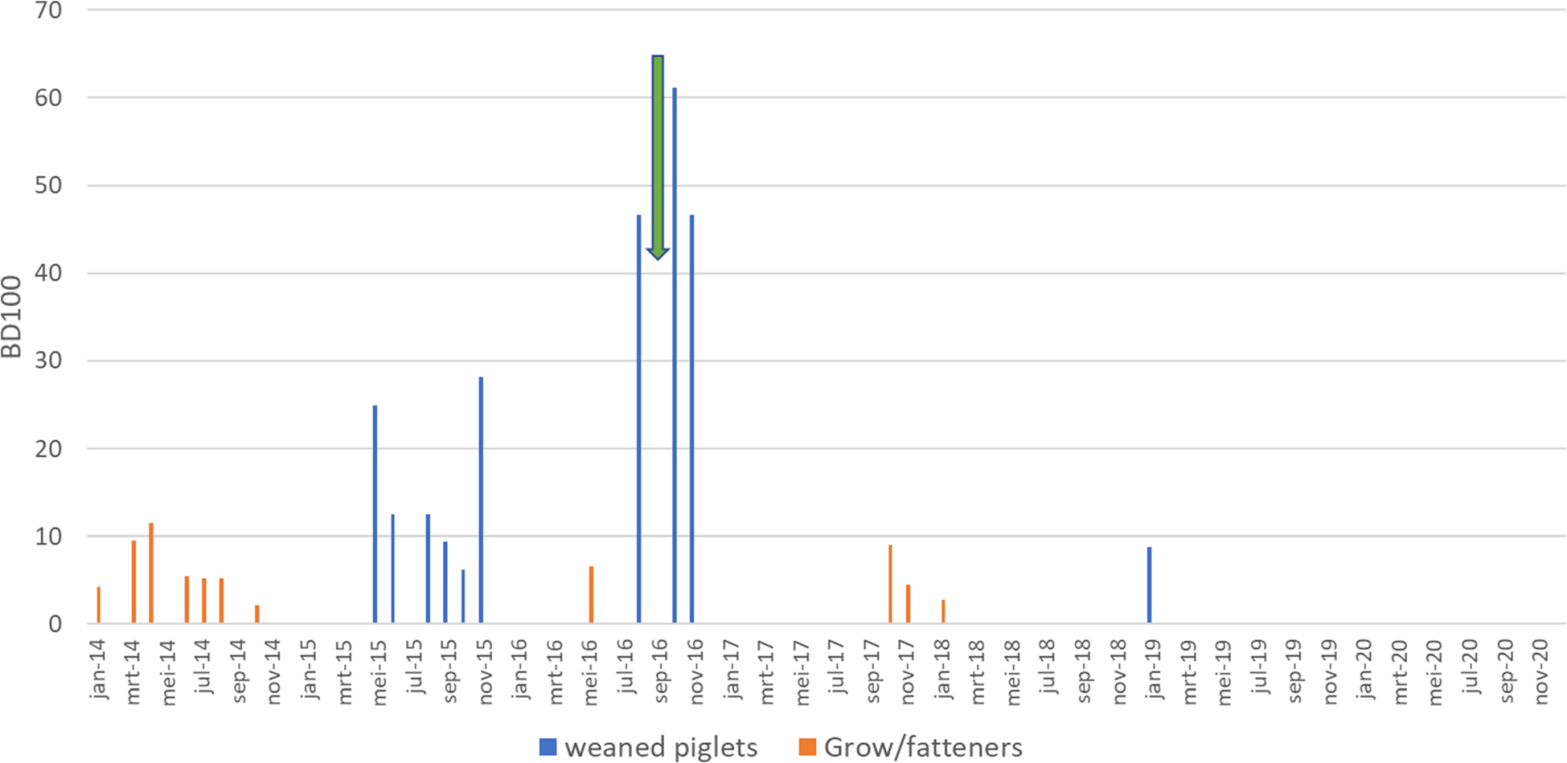
Value of the Belgian antimicrobial treatment index (BD100, AMCRA) for colistin per month for treatment of clinical signs of salmonellosis in weaned piglets and grow/fatteners during the period January 2014 to December 2020 inclusive

**Fig. 3:**
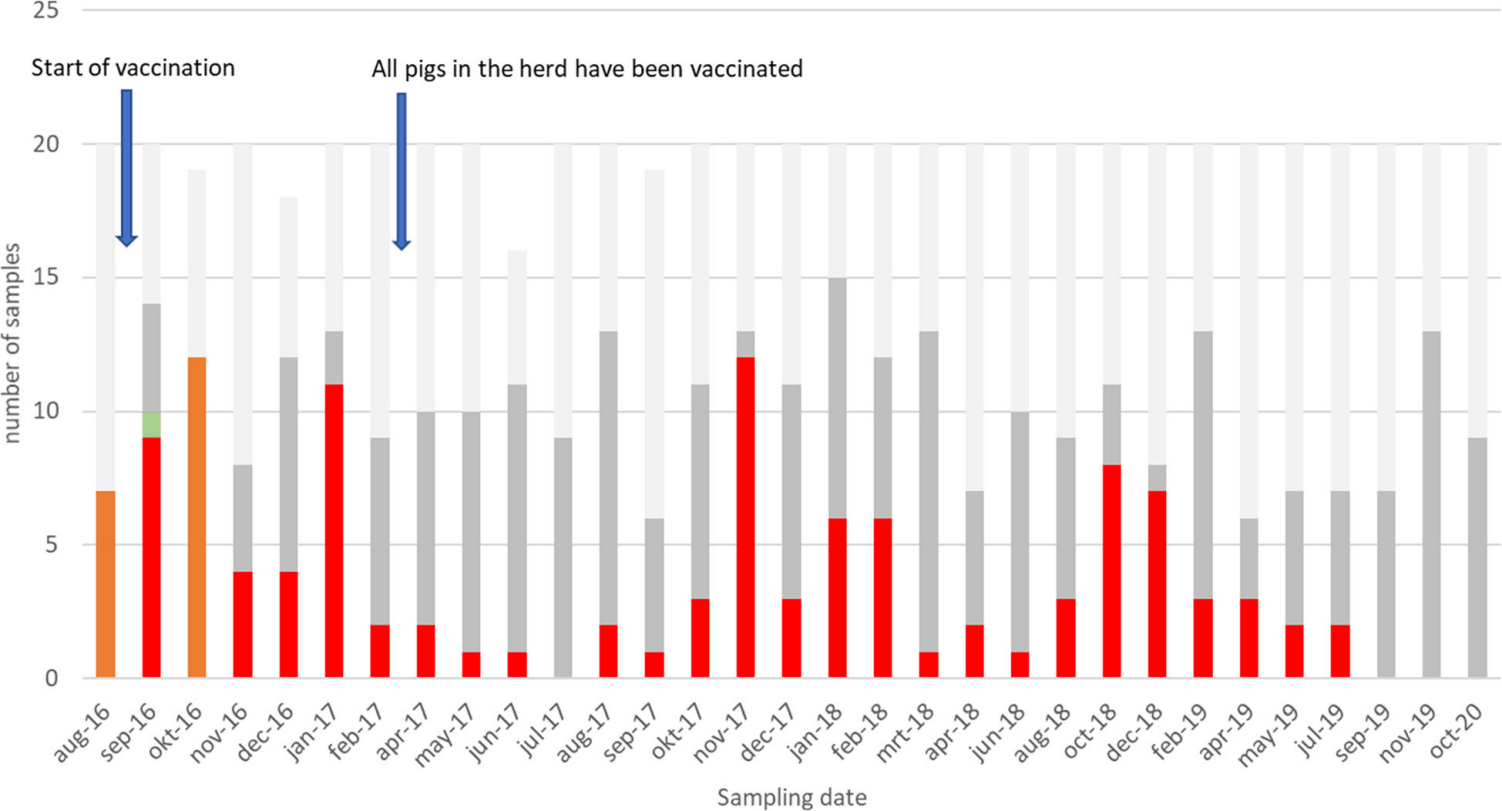
Number of samples taken per sampling date in the period August 2016 to October 2020 differentiated by bacteriological testing result. Dark red bars: *Salmonella* Typhimurium not differentiated between field and vaccine strain, bright red bars: *Salmonella* Typhimurium field strains, green bar: *Salmonella enterica* serogroup C isolate, dark grey bars: Salmoporc® vaccine strain, light grey bars: negative samples. Start of vaccination in August 2016, last non-vaccinated fatteners have been delivered to the slaughterhouse in February 2017

Soon after the first vaccinated animals entered the nursery and subsequently the grow-finishing compartments, the clinical symptoms were reduced to a minimum and hardly any antimicrobial use was necessary (Fig. 2). The number of positive samples decreased significantly during the summer of 2017, compared to the fall of 2016 and January 2017 (Fisher exact test, *P* < 0.05, 11 out of 20 versus 1 out of 20)(Table 3, Fig. 3). However, during fall 2017 there was a period of increased diarrhoea in one compartment of finishers and gilts with a corresponding significant increase in *Salmonella* positive samples from several different locations (Fisher exact test, *P* < 0.05, 3 out of 20 versus 12 out of 20). After this period, the number of positive samples significantly decreased again during the spring and summer of 2018 (March 1/20, April 2/20 and June 1/20 positive samples)(Fisher exact test, *P* < 0.05). During fall and winter 2018/2019 the number of positive samples significantly increased again (Fisher exact test, *P* < 0.05, 1 out of 20 versus 8 out of 20). However, very few clinical signs were observed and only minimal use of colistin sulphate was necessary (Fig. 2, Table 3, Fig. 3). During spring and summer 2019 the number of positives significantly decreased again to finally 0 positive samples in September and November 2019 and October 2020 (Fisher exact test, *P* < 0.05, 8 out of 20 versus 0 out of 20).

### Bacteriological samples

A total of 612 samples were collected in the period from August 2016 to October 2020 over 31 samplings (Table 3, Fig. 3). In five of these, including the last four, no *Salmonella* field strains could be detected within compartments, although on two locations (corridors C2 and C3, September 2019) Salmonella field strains were still found (Table 3). The last three samplings were completely negative for *Salmonella* field strains.

In total 119 *Salmonella* field strains could be isolated of which 99 were *Salmonella* Typhimurium and one isolate was from *Salmonella enterica* serogroup C which was not typed further to serotype level (September 14th, 2016, Table 3). However, 19 isolates were *S.* Typhimurium which were not differentiated as field or vaccine strain. Twelve of these strains isolated on October 25th, 2016 could have been the vaccine strain, because the laboratory did not carry out the differentiation test. Salmoporc® vaccine strain was isolated in 197 samples, mostly from weaned piglets (Table 3).

A total of 86 pairs of sock and dust samples from corridors were collected. In 43 pairs both were negative, in 15 pairs both were positive, in 18 pairs only the sock and in 10 pairs only the dust was positive. The difference in the number of pairs in which either sock or dust was positive was not statistically significant (Chi-square, *P* > 0.05). However, taking the dust sample in addition to the sock sample increased the prevalence by 11.6% from 38.4 to 50%, thereby increasing the detection rate of the sampling method by more than 30%. In the beginning of the trial 48 combinations of sock and dust samples were collected in compartments. In 19 cases both were positive and in 8 cases both were negative. In 13 cases only the sock sample was positive and in 8 cases only the dust sample was positive. The difference in the number of pairs in which either sock or dust was positive was not statistically significant (Chi-square, *P* > 0.05). However, by no longer collecting the dust sample in compartments, the prevalence dropped from 83.3 to 66.7% for field and vaccine strains combined, or from 41.7 to 35.4% for field strains only, thereby decreasing the detection rate of the sampling method by 15%. In 11 samplings in compartments in addition to a sock and dust sample, a pooled faecal sample was collected. In 3 cases all samples were negative and in 1 case all were positive for field strain. In one case a field strain was found in the pooled faecal sample when in both the sock and the dust sample a vaccine strain was found, therefore finding 7 compartments to be positive, instead of 6. This is an increase of the detection rate by 9%. In one case the sock sample and the pooled faecal sample were positive, but the dust was negative. In one sample only the dust was positive. In 4 cases the pooled sample was negative when the sock and dust samples were positive. Comparing sock and pooled samples (*N* = 12, Table 3), 5 sock samples were positive (1 field, 1 vaccine and 3 ST) where the pooled sample was negative, thereby increasing the chance of finding a Salmonella (field, S. Typhimurium or vaccine) from 3/12 to 8/12 using sock samples (Fisher exact test, *P* = 0.0995).

In total 74 S. Typhimurium field strains were isolated in compartments housing pigs. The majority (56/74, 75.7%) were found in rearing gilts / finishers compartments, 7% (5/74) in farrowing compartments, 7% (5/74) in the gestating sow compartment and the AI centre, and 10.8% (8/74) in weaned piglets’ compartments. The samples of the herd visit of the 28th of May 2019 were split and one part was sent to AniCon Laboratory (Anicon Labor GmbH, Höltinghausen, Germany), who ran a multiplex PCR as part of the validation of the test kit (Kylt® *Salmonella* Typhimurium DIVA Real-Time PCR, www.kylt.eu). The results were the same as from the microbiological lab in Hannover detecting no simultaneous presence of field and vaccine strains in the same samples.

## Discussion

This case describes a pig herd which experienced outbreaks of clinical salmonellosis caused by *Salmonella* Typhimurium in weaned piglets, growers, breeding gilts and finishers, which started vaccination with a live attenuated *S.* Typhimurium vaccine (Salmoporc®) in August 2016 in sows, piglets and replacement gilts. Other previously tried interventions, such as strict all-in/all-out, cleaning and disinfection, medication and adding organic acids to the feed and/or drinking water, did not improve the clinical situation. These outbreaks were mostly in finisher pigs and characterised by diarrhoea, loss of appetite during approx. one week, loss of bodyweight and in some cases up to 2% mortality. A previous case report demonstrated that clinical outbreaks of salmonellosis can cost up to € 4.60 per pig sent to slaughter during a 3 month period in which the outbreak occurred [8]. Extrapolated to the herd in this study, this means a loss of approximately € 17,000.- net income in 2015 due to salmonellosis. Vaccinating the pigs at this herd costs about € 15,000.- per year, not counting the costs of labour, saved antimicrobial costs and the psychological burden of having a serious pig disease in your farm. In the long term, the aim would be to have no more *Salmonella* in the weaned piglets and grow/finishers and being able to stop vaccinating the piglets. Vaccinating only the sows and gilts is only a fraction of the costs, but can be considered as an insurance premium against re-emerging or re-introduction of *Salmonella* Typhimurium by e.g. introduction of breeding stock or visitors. The outbreaks in our study herd were treated with colistin sulphate, which is now considered an antimicrobial reserved for last resort treatment in humans and can only be used in pigs under strict regulation [38]. Although clinically effective for the treated batches of pigs, an increasing amount of colistin was needed to treat clinically sick pigs (Fig. 2) to a point were weaned piglets were treated more than 60 out of 100 days in October 2016. This is not a sustainable situation from an animal welfare point of view, a financial perspective of the farmer and the perspective of emerging antimicrobial resistance against polymyxins [39–41]. Because antimicrobial therapy only reduces shedding once treatment of clinically sick pigs has started, untreated pigs can shed *Salmonella*, resulting in rapid spread to other compartments and corridors of the farm. Rapid spread was seen in this herd after the clinical outbreak in the fall and winter of 2017/2018 and 2018/2019, even though clinical signs might be limited to only one or two compartments and at a level where the farmer and the veterinarian didn’t find antimicrobial treatment necessary (Figs. 2 and 3, Table 3). As soon as vaccinated piglets, growers and finishers entered the barns, the clinical situation improved and as a result the antimicrobial use decreased dramatically, which is illustrated in Fig. 2. The number of *Salmonella*-positive samples significantly decreased in spring and summer of 2017, 10 months after the start of the vaccination, from up to 11 S. Typhimurium field strain positive samples to no positive samples in July 2017 and no S. Typhimurium field strain found in the compartments with gilts / finishers in May, June and July 2017 (Table 3). Such a fast improvement after the start of the vaccination has been described before [30, 31]. However, one negative sampling cannot be considered as sufficient proof that the prevalence is low enough to stop vaccinating piglets. Therefore the vaccination and sampling were continued, until October 2020, showing that the significant increases of the number of positive samples in the fall and winter of 2017/2018 and 2018/2019, did not repeat itself in the fall of 2019 and 2020.

Three different sampling schemes (I, II, III) were used for collecting pooled faecal, sock and dust samples, because the sampling scheme was improved during this study based on increased knowledge about the capacity of different sample types to detect *Salmonella* in a pig herd environment, including the results of this study [36]. The dust samples had very little added value in compartments where animals were present (< 5% more positive compartments), however, they do have added value in corridors, which tend to have very little faecal contamination on the floor. Therefore, we decided to use only sock samples in compartments where animals were housed and combined a sock and dust sample in corridors in the final Scheme III. This way we could sample more compartments and corridors with the same total number of samples (17 instead of 10 for a total number of 20 samples), increasing the detection rate of the sampling, and putting more emphasis on sampling compartments of weaned piglets and compartments of growers and finishers and/or rearing gilts.

The possible explanation that sock samples were found to be more sensitive than pooled faecal samples (not only based on the results of this farm) is probably because when using a sock, much more surface is covered and therefore more individual droppings are being sampled. This method increases the chance of sampling a dropping from a pig excreting *Salmonella*. A pooled faecal sample probably contains more faecal material than a sock, thereby increasing the chance of finding *Salmonella* when present [42], but apparently this is overcompensated by the chance of sampling a positive dropping. Also, using pre-enrichment and selective enrichment during culture, makes it possible to detect the presence of very small numbers of *Salmonella* in a sample [43, 44].

Using the described microbiological method, it is not possible to infer anything about the number of *Salmonella* bacteria present in the sample. The use of a semi-quantitative method, like for instance real time PCR, might be able to provide information about the number of *Salmonella* bacteria present in the sample. This (semi) quantitative test could provide information about the level of shedding of *Salmonella*, for example very low in sows which have been vaccinated or very high in clinical situations, as noted by Davies et al. [30] or Jensen et al. [45]. This would give more information about the true status of the herd. These are possible subjects for further study.

In several samplings where we didn’t find field strains of *S.* Typhimurium, we did find vaccine strain. We cannot rule out the possibility that in samples where both field and vaccine strains were present, a colony of vaccine strain was picked for typing instead of a colony of field strain, since only one colony per sample was typed. When more colonies are picked, several serotypes can be found within one sample [46]. Alternatively, a multiplex PCR that can detect simultaneously the DNA of field and vaccine strain, next to general Salmonella spp. DNA, would also solve this problem (e.g. Kylt® *Salmonella* Typhimurium DIVA Real-Time PCR, www.kylt.eu). The vaccine strain can be found in the environment for between 1 and 2 months after stopping vaccination (field data from another farm), however, it is not clear whether this is survival per se or continued shedding by vaccinated animals, which has been shown for 6 weeks after vaccination in experimental setting.

Serology as an additional tool to monitor the *Salmonella* status was not performed, because the current *Salmonella* LPS-ELISA’s cannot differentiate between antibodies due to vaccination or field infection, making interpretation of the results impossible [26]. Differentiation has been done before by Selbitz et al. [35], but these tests were not available to us. Piglets orally vaccinated with Salmoporc® will become serologically positive for a few weeks, but would be serologically negative at slaughter, if no boostering as a result of infection with field strains would take place [47]. Theoretically, boostering as a result of additional contact to vaccine strain at a later age might also be possible, if internal biosecurity between age categories is not optimal, as we found in this study where we found vaccine strain in barn C repeatedly (Table 3). On a national level (e.g. DK, DE and NL [48–50]), general LPS-ELISA’s are used to monitor and categorize pig herds for *Salmonella*. In those countries the inability to differentiate antibodies caused by vaccination and/or infection might lead to problems with such a categorization, however, under the current circumstances where vaccination is not obligatory, only herds in categories II or III would consider vaccination and they are in the risk categories to begin with. Collecting mesenteric lymph nodes at the slaughterhouse might have been an additional method to check for the presence of field strain Salmonella, because pigs can carry Salmonella in their lymph nodes without shedding Salmonella. However, contamination and infection during the period of transport and holding at the slaughterhouse [51] would make sequencing of isolates necessary to differentiate herd strains from those acquired after leaving the farm, which is very labour intensive and costly. Additionally, it has already been shown that vaccination significantly reduces the infection of internal organs including gut associated lymph nodes [47], resulting in a reduced number of contaminated mesenteric lymph nodes at slaughter as a result of vaccination [35]. Finally, any carriers that might start shedding again would have been found during the next sampling, as we found in the fall and winter of 2017/2018 and 2018/2019. We were specifically interested in the Salmonella contamination of the herd environment. *Salmonella* can survive for a very long time, up to 50 months in the environment, for example in slurry [52] or dust [53, 54]. This means that compartments and corridors that are not thoroughly cleaned and disinfected, can become a source of infection for pigs as soon as they enter these compartments, or as in this study, walk along contaminated corridors. The difficulty of effective cleaning and disinfection is well documented for lairages at slaughterhouses ( [55–57] and transport lorries [58], but this also applies to corridors and compartments within farms [59–62] and this study (Table 3). Common practice in pig farms is not to use power washers in compartments above about 1.5 m height, because of the power plugs, feed, water and power lines, lights and ventilator(s), which are located near the ceiling. The dust that settles on these structures is often contaminated with *Salmonella*, as the results of this study demonstrate. In poultry farms, sampling dust is a generally accepted method to demonstrate contamination with *Salmonella* [53, 63]. This means that pig farms can be heavily contaminated with *Salmonella* by *Salmonella-*shedding pigs. Common cleaning and disinfection procedures are in many cases not enough to get rid of all *Salmonella* [60]. Additionally, compartments are almost never cleaned all at the same time because they are only emptied at the same time if an all-in/all-out management is practiced at barn or even herd level, which was not the case in this study. This means that *Salmonella* contamination can easily be carried from one compartment to another by the farmer, employees, visitors, flies, mice, rats or interconnected slurry pits located below the slats of compartments. This is the reason that *Salmonella’s*, once introduced into a farmhouse, can persist for a long time (at least 3 years for example for monophasic *S.* Typhimurium [64]). Together with the regular introduction of infected animals, this is the main reason for the persistence of *Salmonella* within pig farms. In this particular farm, introduction of infected animals did not occur, however the persistence of contamination within the buildings and pigs is very likely, as no new serotype was found during this study which could be the result of new introductions. We cannot exclude that, after a completely negative sampling, the field strains that were found were new introductions, however, we do not consider this likely. Only part or whole genome sequencing (WGS) would be able to demonstrate whether these were different strains, however, this was not part of this study.

Vaccination with Salmoporc® was used to increase the immunity of pigs and thereby decreasing the chance of infection under the given circumstances. Further, in the event of infection, vaccinated pigs will shed less *Salmonella* and during a shorter period, leading to a lower overall contamination of the environment. By continuing the vaccination for a longer period, the contamination of the environment decreases to a point where *Salmonella* can no longer be detected, such as in this case. Given the severe clinical situation before the start of vaccination and the rapid decline during the summer of 2017 we consider it unlikely that a similar effect could have been achieved without vaccination. The rise in the number of positive samples during the fall and winter of 2017/2018 was probably caused by the farmer who relocated some almost-ready-to-breed gilts to a compartment with heavy finishers. In this compartment a water pipeline broke and a lot of water leaked into the slurry pit, resulting in the manure coming up above the slats of the floor because of which the pigs were standing and lying in the slurry. Consequently some pigs developed diarrhoea and one heavy gilt died. No further diagnostic was performed. This was followed by a temporary peak in the number of positive samples in the herd in November 2017 (12/20, Table 3). This again shows, that in non-cleaned and disinfected areas of the farm (underneath the slatted floor) *Salmonella* can survive for a long time and non-expected breakdowns can lead to recontamination. The lack of internal biosecurity protocols, including application by the farmer himself, probably assisted the *Salmonella* spread to the corridors (Table 3) and other compartments. Soon after the issues were solved, the number of positive samples dropped again to only one or two positive samples in the samplings in March, April and June 2018. However, again in fall and winter 2018/2019 the number of positive samples rose to 8 and 7 positives out of 20 each in October and December 2018 respectively. No clear clinical signs were observed, but some diarrhoea was seen and some colistin sulphate was used to treat the diarrhoea symptoms (Fig. 2, Table 3). No clear explanation why the number of positive samples rose in this fall and winter was found.

Salmoporc® vaccine strain has been found in almost all samplings during this study. Because we took sock and dust samples, we cannot say whether this is spilt vaccine or if this was coming from orally vaccinated animals. This result corresponds with results found by Peeters et al. 2019 [28]. In our opinion this does not constitute a public health risk, because the vaccine strain is considered a level 1 biological by the central committee for biological safety in Germany (Zentrale Kommission für biologische Sicherheit (ZKBS)), and therefore safe for humans and animals, also due to its stable double attenuation. Extensive screening of approximately 14,000 human isolates of *Salmonella* Typhimurium by the German reference institute for *Salmonella* (Robert Koch Institute) in the period from 2002 to 2016 showed only one isolation of the vaccine strain, which was the result of accidental self-injection, despite extensive use of this vaccine in pigs in Germany.

Attempts have been made to control *Salmonella* in the pig industry, for example the Danish control program [48], the Belgian control program by Royal decree, UK ZAP program [65], the German QS system [49], but some were stopped due to lack of improvement (UK ZAP program and Belgian program) or showed very slow progress [66] (https://www.q-s.de/pressemeldungen/anzahl-betriebe-erhoehtes-salmonellenrisiko-gering.html). However, none of these included the use of vaccination as a means of control. In Denmark the strategy is now to control this risk at the slaughterhouse during the slaughter process [67]. Cargnel et al. [68] come to the same conclusion, however assuming that vaccination with a live vaccine only reduces the colonisation and excretion of vaccinated animals by 29.96%. Several longer term studies and this study did show that the overall prevalence of *S*. Typhimurium went down to a very high extent [30, 31]. Together with the results of this study, this gives a strong indication that, as in poultry, adding vaccination to the Salmonella control programs might result in a reduction of the number of farms ‘at risk’ and ultimately reduce the number of infections in consumers of pork and pork products.

## Data Availability

The datasets used and/or analysed during the current study are available from the corresponding author on reasonable request.

## Abbreviations

AI: Artificial Insemination
AMCRA: Knowledge centre for antimicrobial use and resistance in animals, www.amcra.be
Approx.: Approximately
CODA-CERVA: Veterinary and Agrochemical Research Centre, Belgium
DGZ: Diergezondheidszorg Vlaanderen (Animal Health Service, Belgium)
ml: Millilitre
Pos: Positive
S: *Salmonella*
ST: *Salmonella* Typhimurium
S Typh: *Salmonella* Typhimurium
Neg: Negative
